# Gene Expression Signatures Predict First-Year Response to Somapacitan Treatment in Children With Growth Hormone Deficiency

**DOI:** 10.1210/clinem/dgad717

**Published:** 2023-12-08

**Authors:** Terence Garner, Peter Clayton, Michael Højby, Philip Murray, Adam Stevens

**Affiliations:** Division of Developmental Biology and Medicine, Faculty of Biology, Medicine and Health, University of Manchester and Manchester Academic Health Science Centre, Manchester, M13 9WL, UK; Division of Developmental Biology and Medicine, Faculty of Biology, Medicine and Health, University of Manchester and Manchester Academic Health Science Centre, Manchester, M13 9WL, UK; Department of Paediatric Endocrinology, Royal Manchester Children's Hospital, Manchester, M13 9WL, UK; Novo Nordisk, Clinical Drug Development, 2860 Søborg, Denmark; Division of Developmental Biology and Medicine, Faculty of Biology, Medicine and Health, University of Manchester and Manchester Academic Health Science Centre, Manchester, M13 9WL, UK; Department of Paediatric Endocrinology, Royal Manchester Children's Hospital, Manchester, M13 9WL, UK; Division of Developmental Biology and Medicine, Faculty of Biology, Medicine and Health, University of Manchester and Manchester Academic Health Science Centre, Manchester, M13 9WL, UK

**Keywords:** predictive markers, growth hormone deficiency, long-acting growth hormone, somapacitan, pretreatment blood transcriptome, and RNA sequencing

## Abstract

**Context:**

The pretreatment blood transcriptome predicts growth response to daily growth hormone (GH) therapy with high accuracy.

**Objective:**

Investigate response prediction using pretreatment transcriptome in children with GH deficiency (GHD) treated with once-weekly somapacitan, a novel long-acting GH.

**Methods:**

REAL4 is a randomized, multinational, open-label, active-controlled parallel group phase 3 trial, comprising a 52-week main phase and an ongoing 3-year safety extension (NCT03811535). A total of 128/200 treatment-naïve prepubertal children with GHD consented to baseline blood transcriptome profiling. They were randomized 2:1 to subcutaneous somapacitan (0.16 mg/kg/week) or daily GH (0.034 mg/kg/day). Differential RNA-seq analysis and machine learning were used to predict therapy response.

**Results:**

121/128 samples passed quality control. Children treated with somapacitan (n = 76) or daily GH (n = 45) were categorized based on fastest and slowest growing quartiles at week 52. Prediction of height velocity (HV; cm/year) was excellent for both treatments (out of bag [OOB] area under curve [AUC]: 0.98-0.99; validation AUC: 0.83-0.84), as was prediction of secondary markers of growth response: HV standard deviation score (SDS) (0.99-1.0; 0.75-0.78), change from baseline height SDS (ΔHSDS) (0.98-1.0; 0.61-0.75), and change from baseline insulin-like growth factor-I SDS (ΔIGF-I SDS) (0.96-1.0; 0.85-0.88). Genes previously identified as predictive of GH therapy response were consistently better at predicting the fastest growers in both treatments in this study (OOB AUC: 0.93-0.97) than the slowest (0.67-0.85).

**Conclusion:**

Pretreatment transcriptome predicts first-year growth response in somapacitan-treated children with GHD. A common set of genes can predict the treatment response to both once-weekly somapacitan and conventional daily GH. This approach could potentially be developed into a clinically applicable pretreatment test to improve clinical management.

Growth hormone deficiency (GHD) is characterized by reduced circulating GH levels. Children with GHD exhibit diminished growth and adult height, and reduced quality of life in terms of physical functioning, and emotional and social well-being (1, 2). For decades, GH replacement therapy in children with GHD has involved daily subcutaneous injections of recombinant human GH to improve overall health and adult height (3, 4). Due to a complex variety of factors, response to GH therapy is variable and not always successful. This is problematic since treatment is costly and daily GH injections are burdensome for patients and their caregivers, disrupting and interfering with daily life. The treatment burden associated with daily subcutaneous injections is at least partly responsible for suboptimal clinical outcomes resulting from relatively high nonadherence to prescribed replacement therapy in the real world (5, 6).

Novel long-acting GH (LAGH) formulations aim to establish a less frequent, and therefore less burdensome, dosing regimen that interferes less with daily life and provides the same excellent efficacy and safety profile as GH administered daily to potentially improve adherence and clinical outcomes (5). For instance, the LAGH somapacitan is a human GH derivative (99% similar to endogenous human GH) with a single amino acid substitution linked to a short noncovalent reversible albumin-binding moiety to delay its elimination, prolong its half-life, and extend its duration of action to allow once-weekly administration (7). Somapacitan was recently approved for once-weekly GH replacement therapy in adults and children with GHD and is currently in phase 3 development for treating short stature in 4 nonreplacement indications: children born small for gestational age (8), Turner syndrome (TS), Noonan syndrome, and idiopathic short stature (REAL8, NCT05330325; REAL9, NCT05723835).

Despite potentially better clinical outcomes with GH treatment using novel LAGHs (9), growth responses are known to vary from patient to patient on GH therapy (10). Predicting growth response to GH therapy is an ongoing challenge as it may be influenced by age/puberty stage, environment, genetic variants (which may impact drug absorption, distribution, metabolism, and excretion), mechanism of action of the drug, and/or disease etiology. Most prediction models implementing either clinical measurements or the patient genetic background have had little success in practice due to the influence of covariates related to the developmental stage of the child, the severity of the disease, and geographical location (11, 12). Existing prediction tools based on linear regression models using patient characteristics and clinical phenotype account for only about 60% of the variance in response to GH treatment for children with GHD (13). Transcriptomics represents a promising diagnostic and predictive tool for response to therapy since it simultaneously captures information about genetics, developmental stage in relation to age, and the impact of the local environment on gene expression patterns (14). Indeed, pretreatment blood gene expression data acquired from microarrays coupled with random forest algorithm analyses are demonstrated to be useful for (1) diagnosing childhood GHD (15) and (2) predicting growth response for children with GHD and children with TS receiving daily GH treatment (16).

Here, we perform peripheral whole blood transcriptome profiling using next-generation RNA sequencing (RNA-seq) as the source of pretreatment gene expression profiles for treatment-naïve prepubertal children with GHD enrolled in the REAL4 study. We show that common patterns of gene expression detected before initiating treatment can be used to predict the first-year response in children with GHD receiving either LAGH treatment with once-weekly somapacitan or conventional treatment with daily GH.

## Materials and Methods

### Patients

The REAL4 study (ClinicalTrials.gov: NCT03811535) is an ongoing randomized, multinational, open-labelled, and active-controlled parallel-group phase 3 trial. The study was conducted in Austria, Canada, France, Germany, India, Israel, Italy, Japan, Korea, Latvia, Poland, Russia, Serbia, Slovenia, Spain, Switzerland, Thailand, Ukraine, United Kingdom, and United States. The sponsor (Novo Nordisk A/S) designed the trial and oversaw its conduct. The main trial period was 52 weeks (data collected between May 2019 and November 2021), followed by an ongoing 3-year single-group extension period. Trial design, eligibility, key inclusion/exclusion criteria, patient demographics, and clinical results following 52 weeks of treatment have been previously reported (17). Briefly, 200 GH treatment-naïve prepubertal children with GHD (between 2.5 and 10 years of age for girls and 2.5 and 11 years of age for boys) were enrolled and randomized 2:1 to receive either once-weekly somapacitan (0.16 mg/kg/week) or daily GH (0.034 mg/kg/day; Norditropin, Novo Nordisk) administered subcutaneously. Randomization was stratified by region (Japan; rest of the world) as well as by age (<6; ≥6 years), sex (boys; girls), and GH peak level (<7.0; ≥7.0 ng/mL). The REAL4 study was approved by local and national ethics committees, as appropriate, and conducted in accordance with the International Conference on Harmonisation Guidelines for Good Clinical Practice (18) and the Declaration of Helsinki (19). Informed consent was obtained in writing from the parents and/or the child's legally acceptable representative, and child assent was obtained as age appropriate.

### Processing of Biological Samples

To assess the pretreatment blood transcriptome, total RNA was isolated from whole-blood samples collected at baseline and depleted of globin mRNA using the GlobinClear-Human Kit (Life Technologies # AM1980) before being converted into cDNA libraries using the Illumina TruSeq Stranded mRNA sample preparation kit (Illumina #RS-122-2103) at EA | Q2 Solutions (Durham, NC). Final cDNA libraries were quantified, normalized, and pooled before sequencing using HiSeq-Sequencing-2 × 50 bp-PE on an Illumina sequencing platform. Quality control analysis, genome alignment, and gene/isoform quantification was performed after sequencing using the RNAv9 pipeline developed by EA | Q2 Solutions (Durham, NC), which uses a variety of internally developed and open-source programs. Final processing of transcriptomic data via the edgeR package (3.32.1) in R (4.0.3) removed genes that were expressed with fewer than 10 counts per million to generate a final count matrix of 19 039 genes from 121 samples.

### Insulin-Like Growth Factor-I Assay

Insulin-like growth factor-I (IGF-I) is a key pharmacodynamic marker for GH therapy response. IGF-I analyses were performed by a central laboratory using a commercially available assay kit (Immunodiagnostic Systems Immunoassay) on samples collected at weeks 13 and 39 (day 7 after somapacitan dosing for assessing trough levels), weeks 4 and 26 (in a window of 1-4 days after somapacitan dosing designed to characterize the peak), and week 52 (4-6 days after somapacitan dosing to capture expected weekly average IGF-I levels).

### Classification of Growth Response

Growth response was calculated as height velocity (HV; cm growth per year), as previously described (16). Three secondary response measures were also calculated: HV standard deviation score (SDS) (HVSDS) (20), change in height SDS from baseline to week 52 (ΔHSDS) (21), and change in IGF-I SDS from baseline to week 52 (ΔIGF-I SDS) (22). In all cases, children were grouped into quartiles to compare fast and slow responders to growth hormone, separately for those on daily and once-weekly growth hormone. Comparisons were made between the fastest growing quartile and the remaining 3 quartiles and between the slowest quartile and remaining 3.

Clinical variables were compared between groups for the primary growth response measure (ie, HV). Variables were selected based on previous work on prediction of growth response from clinical variables (23). The baseline variables compared were age, sex, HSDS, HV, HVSDS, midparental HSDS, IGF-I SDS, and peak GH level. These data are presented as the mean ± SD and differences between the target quartile and all remaining participants were computed using the Wilcoxon rank-sum tests (Supplementary Material S1 (24)).

### Generation of Predictive Models

Following quality control, alignment and quantification, gene count data were imported to R version 4.3.0 and genes with low expression were removed. Counts were log transformed and normalized to account for library size variations between samples, generating trimmed mean of M-values.

Differential expression was performed, using a generalized linear model quasi-likelihood fit model, to identify genes associated with each growth response metric, separately for daily GH and somapacitan. Using this approach, the top 100 differentially expressed genes (ranked on *P* values) were identified as targets for prediction, generating distinct sets of genes for each analysis. As unbalanced groups can lead to poor prediction of the minority class by machine learning classification algorithms, sample groups were balanced using the synthetic minority oversampling technique (25) prior to each predictive modeling analysis. Subsequently, the feature selection algorithm Boruta (26) was used to identify the genes that were most informative in distinguishing HV quartiles prior to prediction. Varying genes sets were used to predict each metric; complete lists are available (Supplementary Materials S2-S13 (24)) with gene ontology summaries generated using over-representation analysis via WebGestalt (https://www.webgestalt.org/) (27). For assessment of prediction using clinical parameters, models were generated using the same variables compared between response groups (Supplementary Material S1 (24)).

Random forest is a machine learning classification algorithm which summarizes decision trees to identify patterns that can distinguish groups (28). Models of 1000 trees were generated to identify patterns of gene expression that can predict the observed growth response. Predictive quality is defined by the area under curve (AUC) of the receiver operating characteristic curve, which describes the sensitivity and specificity of a test. AUC can be defined on prediction in an independent set of samples withheld for validation. If the sample set is not sufficiently large to generate an independent validation set, out of bag (OOB) measures represent the best validation available within a dataset with limited samples. As only a subset of samples are selected for any individual decision tree to train a random forest model, OOB measures utilize those unselected samples to test the accuracy of the model being trained.

For children receiving once-weekly somapacitan, sufficient samples were available to perform an internal validation by splitting samples into training (70% of samples) and validation (30% of samples) sets. In addition to this, OOB AUCs were generated for all samples to ensure direct comparisons were possible between somapacitan and daily GH treatments.

### Statistics

Baseline characteristic values and 52-week results for efficacy and IGF-I SDS within the transcriptomics cohorts were performed as previously described (17). Descriptive statistics for HV and changes from baseline to week 52 in HVSDS, HSDS, and IGF-I SDS within the somapacitan and daily GH transcriptome cohorts in REAL4 are presented here.

## Results

### Clinical Phenotype of Participants With Transcriptomic Data Available

**Figure 1.**
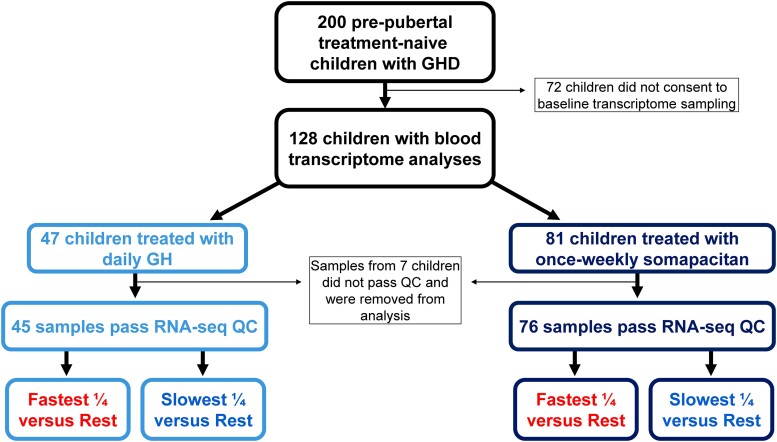
Pretreatment blood transcriptome study design for REAL4. Schematic representation of pretreatment blood transcriptome cohorts from the REAL4 study, study design, and transcriptomics workflow. Prepubertal, treatment-naïve children with GHD were treated with daily GH or once-weekly somapacitan for 52 weeks. Abbreviations: GH, growth hormone; GHD, GH deficiency; RNA-seq, RNA sequencing; QC, quality control.

A total of 128 prepubertal, treatment-naïve children with GHD from the REAL4 trial consented to baseline blood transcriptome profiling by RNA-seq (somapacitan, n = 81; daily GH, n = 47). Across all sequenced samples, the median number of actual sequenced reads was 46.8 million, with 42.9 million on-target reads after removing various sequencing artifacts. The samples had a median of 16 784 genes and 37 578 isoforms detected. Transcriptome data for 121 patients passed quality control (somapacitan, n = 76; daily GH, n = 45), with the remaining 7 samples removed from further analyses (Fig. 1).

**Figure 2.**
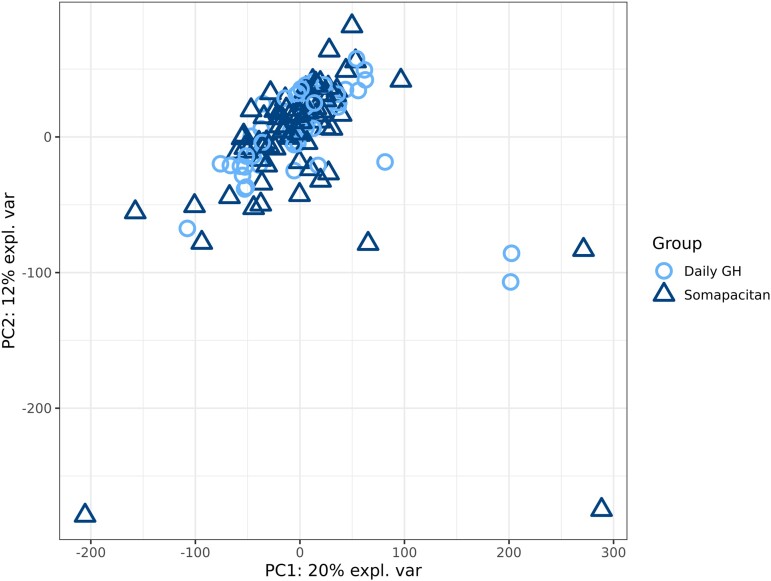
Principle component analysis of pretreatment blood transcriptome. Principle component (PC) analysis demonstrates that pretreatment blood transcriptome variation is not dependent on treatment group. Each point represents a summary of all genes in the entire blood transcriptome (n = 19 039) for a participant (n = 121). Dark triangles and light circles distinguish which arm of the study participants were subsequently assigned (somapacitan or daily GH, respectively).

Demographics and baseline characteristics for the cohort of patients with available transcriptomic data were largely similar in both treatment groups, although—as observed with the full REAL4 population (full analysis set; FAS) (17)—mean HV, HSDS, HVSDS, IGF-I SDS, and GH peak levels at baseline were slightly lower numerically in the control daily GH group than in the once-weekly somapacitan treatment group (Table 1). Efficacy results and change from baseline in IGF-I SDS at week 52 were similar between treatment groups (Table 2) and align well with published results from the full REAL4 population (FAS) demonstrating once-weekly somapacitan has a similar efficacy and safety profile as daily GH with similar mean IGF-I levels after 52 weeks of treatment (17). A comparison of clinical parameters relevant to predicting growth response reveals expected variation between response groups. Expected associations between age, midparental height, GH level, and growth were observed (Supplementary Material S1 (24)). Importantly, we found no evidence of confounding relationships between clinical phenotypes and transcriptome, indicating transcriptome variation is not dependent on treatment group (Fig. 2).

**Table 1.** Baseline characteristics

	Somapacitan transcriptome cohort n = 76/132	Daily GH transcriptome cohort n = 45/68	Somapacitan FAS n = 132	Daily GH FAS n = 68
Mean age, years (SD)	6.2 (2.3)	6.5 (2.4)	6.4 (2.2)	6.4 (2.4)
<6 years, n (%)	42 (55.3)	22 (48.9)	64 (48.5)	33 (48.5)
Female, n (%)	21 (27.6)	12 (26.7)	33 (25.0)	18 (26.5)
Race, n (%)				
White	50 (65.8)	28 (62.2)	78 (59.1)	36 (52.9)
Asian	21 (27.6)	15 (33.3)	46 (34.8)	28 (41.2)
Black or African American	0 (0)	1 (2.2)	0 (0)	1 (1.5)
Not reported	1 (1.3)	0 (0)	7 (5.3)	3 (4.4)
Other	4 (5.3)	1 (2.2)	1 (0.8)	0 (0)
Mean weight, kg (SD)	16.3 (4.63)	16.3 (4.64)	16.7 (4.60)	16.0 (4.95)
Mean BMI, kg/m^2^ (SD)	15.6 (1.56)	15.7 (1.44)	15.7 (1.59)	15.6 (1.38)
Mean height, cm (SD)	101.1 (12.6)	101.0 (13.6)	102.3 (12.5)	100.2 (15.0)
Mean HV, cm/year (SD)	4.6 (1.3)	4.0 (1.5)	4.3 (1.4)	4.1 (1.4)
Mean HVSDS (SD)	−2.16 (1.37)	−2.53 (1.47)	−2.35 (1.51)	−2.52 (1.55)
Mean HSDS (SD)	−3.02 (0.92)	−3.31 (1.26)	−2.99 (1.02)	−3.47 (1.52)
Mean IGF-I SDS (SD)	−2.01 (0.98)	−2.39 (1.02)	−2.03 (0.97)	−2.33 (1.03)
GH peak, µg/L (SD)	4.83 (2.41)	4.45 (2.86)	4.93 (2.50)	4.10 (2.77)
Etiology, n (%)				
Idiopathic	67 (88.2)	39 (86.7)	115 (87.1)	61 (89.7)
Organic	9 (11.8)	6 (13.3)	17 (12.9)	7 (10.3)

Abbreviations: BMI, body mass index; FAS, full analysis set; GH, growth hormone; HSDS, height standard deviation score; HV, height velocity; HVSDS, height velocity SD score; IGF-I SDS, insulin-like growth factor-I SD score.

**Table 2.** Efficacy and pharmacodynamic results for transcriptome cohorts after 52 weeks of treatment

	Somapacitan transcriptome cohort n = 76	Daily GH transcriptome cohort n = 45
Mean (SD) HV, cm/year	11.3 (2.6)	11.7 (2.3)
Mean (SD) HV SDS	5.60 (2.84)	6.42 (3.00)
Mean (SD) ΔHSDS	1.25 (0.57)	1.31 (0.50)
Mean (SD) ΔIGF-I SDS	2.30 (1.20)	2.35 (0.95)

Abbreviations: Δ, change from baseline to week 52; GH, growth hormone; HV, height velocity; HSDS, height SDS; IGF-I, insulin-like growth factor-I; SDS, SD score.

### Predicting Growth Response in HV to Once-weekly Somapacitan and Daily GH Treatment

The primary efficacy endpoint for growth response in REAL4 was annualized HV after 52 weeks of treatment. To assess good and poor responders to once-weekly somapacitan and daily GH treatments in this study, we split our cohort of patients into quartiles based on their annualized HV at week 52 to identify the fastest (good responders) and slowest (poor responders) growers to each treatment (Fig. 1). The OOB AUC and error rate (ER) for the fastest and slowest growers for once-weekly somapacitan were 0.994 (OOB ER = 3.51) and 0.991 (OOB ER = 7.02), respectively (Table 3). OOB AUCs were also high for the fastest (OOB AUC = 0.984, ER = 7.46) and slowest responders (OOB AUC = 0.99, ER = 3.51) to daily GH treatment. Although there were not sufficient participants in the daily GH group to generate an independent validation set, the somapacitan group of the cohort enabled us to add robustness to these findings with a full internal validation. In this group, we can robustly predict the fastest and slowest responders with validation AUCs of 0.889 and 0.833 (Table 3), respectively.

**Table 3.** Predicting growth response to somapacitan and daily GH using pretreatment RNA-seq or clinical variables

		Somapacitan transcriptome cohort		Daily GH transcriptome cohort	
	Subgroup	Fastest ¼ vs rest	Slowest ¼ vs rest	Fastest ¼ vs rest	Slowest ¼ vs rest
HV (cm/year) prediction from pretreatment RNA-seq analysis	OOB AUC	0.994	0.991	0.984	0.99
	OOB error rate %	3.51	7.02	7.46	3.51
	Validation AUC	0.889	0.833	—	—
HV (cm/year) prediction from clinical variables	OOB AUC	0.922	0.918	0.937	0.986
	OOB error rate %	16.67	11.54	12.24	4.76
	Validation AUC	0.614	0.743	—	—

Abbreviations: Δ, change from baseline to week 52; AUC, area under the curve; GH, growth hormone; HV, height velocity; OOB, out of bag.

To compare the predictive performance of pretreatment blood transcriptome against the predictive performance of clinical variables, we performed the above analysis using clinical variables commonly calculated prior to GH treatment (Supplementary Material S1 (24)). In all cases, OOB AUCs and validation AUCs calculated using the pretreatment blood transcriptome alone outperformed analyses using clinical variables alone (Table 3).

### Predicting Secondary Growth Response Endpoints to Once-weekly Somapacitan and Daily GH Treatment

We next sought to assess the ability of pretreatment blood transcriptome to predict secondary growth response endpoints: HVSDS and ΔHSDS. OOB AUCs were similarly high for HVSDS and ΔHSDS in both the somapacitan and daily GH treatment groups. Prediction in children receiving daily GH yielded OOB AUCs between 0.990 and 0.998 (ERs = 1.49 and 5.26), compared with OOB AUCs between 0.979 and 0.994 in the somapacitan groups (ERs = 4.39 and 5.26) (Table 4). Validation AUCs were lower than for HV, ranging from as low as 0.611 for predicting ΔHSDS for the fastest growers and up to 0.778 for predicting HVSDS for the fastest growers. Taken together, the accuracy of predicting growth response to once-weekly somapacitan treatment in children with GHD was strongest for HV (ie, cm/year), which outperformed the accuracy of predicting secondary growth parameters (HVSDS and ΔHSDS).

**Table 4.** Predicting secondary efficacy and pharmacodynamic endpoints to somapacitan treatment from pretreatment RNA-seq analysis

		Somapacitan transcriptome cohort		Daily GH transcriptome cohort	
	Subgroup	Fastest ¼ vs rest	Slowest ¼ vs rest	Fastest ¼ vs rest	Slowest ¼ vs rest
HVSDS	OOB AUC	0.988	0.990	0.995	0.990
	OOB error rate %	4.39	4.39	4.48	5.26
	Validation AUC	0.778	0.750	—	—
ΔHSDS	OOB AUC	0.979	0.994	0.998	0.995
	OOB error rate %	5.26	4.39	1.49	5.26
	Validation AUC	0.611	0.750	—	—
ΔIGF-I SDS	OOB AUC	0.976	0.960	0.998	0.987
	OOB error rate %	10.11	10.11	3.33	9.80
	Validation AUC	0.885	0.850	—	—

Abbreviations: Δ, change from baseline to week 52; AUC, area under the curve; GH, growth hormone; HV, height velocity; HSDS, height SDS; IGF-I, insulin-like growth factor-I; OOB, out of bag; SDS, SD score.

### Predicting IGF-I Response to Once-Weekly Somapacitan and Daily GH Treatment

IGF-I is the most widely used pharmacodynamic marker of GH response. To complement our analysis of growth response prediction, we therefore explored the ability of pretreatment blood transcriptome to predict IGF-I SDS change from baseline to week 52 (ΔIGF-I SDS). The prediction of ΔIGF-I SDS was high and, on average, similar to HV prediction, albeit with greater error (Table 4). Prediction in children receiving daily GH yielded OOB AUCs of 0.998 and 0.987 for fastest and slowest growers, respectively (ER = 3.33 and 9.80, respectively). In the somapacitan groups, OOB AUCs were 0.976 and 0.960 (both ER = 10.11). Validation AUCs demonstrate that the accuracy of predicting ΔIGF-I SDS for the fastest and slowest responders to once-weekly somapacitan treatment (0.885 and 0.850, respectively) were similar to HV.

### Genes Predictive of Growth Response to Once-Weekly Somapacitan and Daily GH Treatment

Recently, a set of common genes have been found by pretreatment, microarray-based transcriptome analyses to predict daily GH treatment response with high accuracy in children with GHD as well as children with TS (16). To validate those findings, we assessed this set of genes previously identified as being predictive of first year growth response to daily GH treatment and found that they have predictive value for both once-weekly somapacitan (OOB AUC = 0.846-0.930, ER = 17.54-24.56) and daily GH (OOB AUC = 0.667-0.971, ER = 11.94-38.60) treatment groups in the current REAL4 study (Table 5). Interestingly, these genes consistently performed slightly better in predicting the fastest growing children compared with the slowest growing children. Collectively, these results demonstrate a consistency in our ability to predict growth response in children with GHD following treatment with conventional daily GH as well as with once-weekly somapacitan, a novel LAGH alternative.

**Table 5.** Common gene set predicts growth response to daily GH and once-weekly somapacitan

		Somapacitan transcriptome cohort		Daily GH transcriptome cohort	
	Subgroup	Fastest ¼ vs rest	Slowest ¼ vs rest	Fastest ¼ vs rest	Slowest ¼ vs rest
HV, cm/year	OOB AUC	0.971	0.667	0.930	0.846
	OOB error rate %	11.94	38.60	17.54	24.56

Abbreviations: AUC, area under the curve; GH, growth hormone; HV, height velocity; OOB, out of bag.

## Discussion

In the current study, we demonstrate robust first-year growth and IGF-I response predictions for children with GHD treated with a novel LAGH (once-weekly somapacitan). This prediction of fastest and slowest responders was achieved by applying RNA-seq to identify gene expression patterns from a blood transcriptome sample taken prior to therapy initiation. We also demonstrate an internal validation of previous observations made using microarray analyses to predict response to daily GH treatment in children with GHD and TS. Together, these results suggest common gene expression patterns can be used to predict the first-year growth and IGF-I response in children with GHD receiving either conventional daily GH treatment or LAGH treatment with once-weekly somapacitan.

The response to GH therapy is highly variable between individual patients and depends on many factors, including age of initiating treatment, sex, distance to parental height, peak GH level on stimulation testing, and starting dose (4). This variability is particularly concerning given the high financial costs associated with GH treatment and the burden of daily injections for children with GHD and their caregivers. Reducing treatment burden with a LAGH (eg, 313 fewer injections a year when a child with GHD is treated with once-weekly somapacitan) is proposed to improve adherence and potentially lead to better clinical outcomes; however, observed variations in GH replacement response are expected to remain an ongoing challenge. Predictive models of GH therapy response are therefore valuable when guiding treatment decisions, with existing tools modeling clinical phenotype alone in children with GHD accounting for only about 60% of the variance in growth response (13). Although there are no current genomic markers for predicting positive or negative responses, emerging evidence for the polygenic nature of response to GH therapy supports pretreatment whole blood transcriptome profiling as a potentially effective prediction tool (14).

Transcriptome analyses reflect both the genetic profile of a patient as well as the complex clinical phenotypes arising from variations in GHD severity and changes during development. The blood transcriptomic profile of GHD and TS patients has already been shown to be associated with daily GH treatment response and correlate with disease severity (29). More recently, gene expression signatures identified by pretreatment blood transcriptomic profiling were found to predict response to daily GH treatment in GHD and TS (16). Although pretreatment blood transcriptome was found to result in superior GH treatment response prediction when compared with using clinical phenotype markers alone, a significant decrease of error rate was achieved when combining blood transcriptome markers with clinical phenotype markers (16).

Our analyses here confirm these findings and support the analysis of gene signatures obtained from pretreatment blood transcriptomics as an effective predictor of GH treatment response for children with GHD. We demonstrate that pretreatment transcriptome analyses can classify both the fastest and slowest quartiles for growth response (measured in HV after 52 weeks of treatment) with OOB AUC up to 0.99 and 0.98 for somapacitan and daily GH, respectively. Meanwhile, the validation AUCs for growth response prediction to somapacitan were 0.889 and 0.833 for fastest and slowest quartiles, respectively. Additionally, we illustrate that, despite significant differences in clinical parameters between response groups, predictive models using clinical variables alone are outperformed by models using transcriptomics alone. This result suggests that variations in clinical parameters do not bias the results of the transcriptomic random forest, but rather the blood transcriptome itself reflects—to a large extent—the underlying clinical variation.

An identical set of genes previously found to be predictive of daily GH treatment in pediatric GHD and TS were able to predict growth responses with once-weekly somapacitan in GHD (OOB AUC for fastest quartile = 0.93-0.97; OOB AUC for slowest quartile = 0.67-0.85). Taken together, these results represent a major advance toward individualized prediction of response to GH therapy and provide the potential basis for a pretreatment predictive test across indications requiring GH therapy and perhaps also different GH formulations (daily GH vs LAGHs like somapacitan).

Feature selection, performed using Boruta ahead of prediction, refined a unique set of genes most likely to be predictive of response to each individual growth metric, with variable predictive capacity. This implies some difference in the gene expression patterns associated with each metric and the same is true of the different genes identified for daily GH and weekly LAGH. Despite this, there is no indication that the differences in predictive genes are a result of functional differences between treatments, which is supported by the comparable efficacy and safety of the 2 treatments. Further, a limited relationship was observed between HV and the secondary markers of growth response. Previous work has also established that there is high transcriptomic heterogeneity underpinning the relationship between IGF-I and height response following treatment with GH, with significant interindividual variation (30). Here we demonstrate that, despite this heterogeneity, change in IGF-I SDS is associated with a consistent transcriptomic signature, while the population normalized measures of growth response tested, HVSDS and ΔHSDS, are not. The relatively poor prediction of HVSDS compared with HV indicates an as yet unresolved contribution of genetic factors relating age and sex to growth response. This is complicated by the effective prediction of IGF-I SDS, another population normalized measure, suggesting a difference in the effects of age and sex on IGF-I and HV. We note that when comparing to published work on the prediction of GHD severity (31), there is limited overlap with genes determined as predictive of response here. A noted commonality was significant differential expression of the bisphosphoglycerate mutase gene (*BPGM*) in both fast and slow responders treated with once-weekly somapacitan (Supplementary Materials S12 and S13 (24)). This gene has previously been associated with body height in genome-wide association studies (32, 33).

The current work represents a major step toward personalizing the prediction of GH therapy response. The results presented here validate a previous proof of concept study (14) and expands these analyses into the novel LAGH space, showing that gene expression patterns identified from a baseline blood transcriptome sample can predict the first-year growth response in children with GHD receiving either daily GH or once-weekly somapacitan treatment. Future studies will be required to test this approach for other recently developed LAGHs, such as somatrogon and lonapegsomatropin, which each utilize distinct protraction technologies (9). Baseline transcriptome samples are being collected for the recently initiated phase 3 REAL8 study investigating once-weekly somapacitan as treatment for short stature in nonreplacement indications, such as short children born small for gestational age, TS, Noonan syndrome, and idiopathic short stature (NCT05330325). This provides an excellent opportunity in the near future to explore possible disease-specific gene expression patterns and potentially be able to refine common gene expression patterns across diseases to enhance growth response prediction to somapacitan treatment.

Since a common set of genes can be used to classify the growth response to once-weekly somapacitan treatment in children with GHD and daily GH treatment in children with GHD, a transcriptomic test and prediction algorithm could be developed to potentially improve clinical management by offering physicians insights into expected growth responses for individual patients before initiating treatment. Although further validation will be required to develop such a clinical test, a test based on this approach could inform clinicians and guide treatment decisions when presented with, for instance, a patient predicted to be poor responder.

In future work, the prediction of growth and IGF-I response to GH/LAGH treatment could be improved by the integration of causal modeling approaches to infer causal relationships between predictive genes and growth response. This would offer insight into the mechanisms underlying the variable prediction of growth response to therapy and thereby increase the accuracy with which we can predict expected responses for individual patients.

## Data Availability

The datasets generated during and/or analyzed during the current study are not publicly available but are available from the corresponding author on reasonable request.

## Clinical Trial Information

NCT03811535 (registered January 22, 2019).

## Abbreviations

AUC: area under curve
ER: error rate
FAS: full analysis set
GH: growth hormone
GHD: GH deficiency
LAGH: long-acting GH
HV: height velocity
HVSDS: height velocity standard deviation score
HSDS: height standard deviation score
IGF-I: insulin-like growth factor-I
OOB: out of bag
SDS: standard deviation score
TS: Turner syndrome
